# Commentary: Modeling mortality risk in patients with severe COVID-19 from Mexico

**DOI:** 10.3389/fmed.2023.1247741

**Published:** 2023-09-28

**Authors:** Elaheh Sanjari, Sepideh Toosizadeh, Hadi Raeisi Shahraki

**Affiliations:** ^1^Student Research Committee, Shahrekord University of Medical Sciences, Shahrekord, Iran; ^2^Department of Epidemiology and Biostatistics, Faculty of Health, Shahrekord University of Medical Sciences, Shahrekord, Iran

**Keywords:** prediction, regression, variable importance, odds ratio, statistical modeling

## Introduction

We meticulously read the paper by Cortes-Telles et al. (1) that was published online in *Frontiers in Medicine* in May 2023. The study was conducted to determine significant predictors of mortality among hospital-admitted COVID-19 patients. Finally, they ranked the five crucial predictors of death as (1) need to a mechanical ventilator, (2) platelet concentration at admission, (3) increased derived neutrophil to lymphocyte ratio, (4) age and (5) pulse oximetry saturation respectively (1). Undoubtedly, their study makes a valuable contribution to the area, but some methodological issues need to be taken into account to avoid misinterpretation of the study's results.

## The higher OR doesn't necessarily show the best predictor

The odds ratio is a valid metric to investigate any association between the quantitative independent variables and a binary outcome but the presence of such an association has no information about the prediction capability. The OR is affected by variables' scales and may not be comparable due to the fact that they have different types of units. Instead, the standardized ORs that are extracted from the standardized regression coefficients have the same unit and are comparable (2). Moreover, to compare the prediction accuracy of models, the area under the cure (AUC) is highly suggested (3).

## LASSO is not appropriate for explanation modeling

Using regression models for the causal explanation is very different from the empirical prediction aims. If highly correlated variables exist, the lasso retains only one variable and sets the others to zero. That will possibly lead to misleading results for the explanation aims. So, like all greedy algorithms, LASSO is good for prediction aims and not appropriate for explanation aims. More information about the difference between the explanation and prediction models, reading an article entitled “to explain or to predict” is helpful (4).

## The presence of sparse data bias

The lack of adequate case numbers for some of the variables in the logistic regression leads to a phenomenon called sparse data biased. A further upward bias is expected due to the fact that odds are obtained by taking the exponentiation of the coefficients which leads to impossibly huge odds (5). Cortes-Telles et al. (1) report the need for a mechanical ventilator equal to 193 (43 to 878) and the logarithm of platelets counts as 0.002 (0.0003 to 0.09) and the logarithm of dNLR as 14.1 (1.2 to 169.5) which are not reliable.

## Discussion

The take-home message of this note for the readers is that using true statistical analysis and an appropriate interpretation is critical in medical investigations. To avoid sparse data bias, using Firth's bias-reduced logistic regression which uses penalized maximum likelihood estimation, the exact logistic regression and Bayesian approaches are recommended.

## Author contributions

ES, ST, and HR: conception and design and drafting the article. Also, the manuscript has been read and approved by all the authors. All authors contributed to the article and approved the submitted version.
